# The left-lateralized N170 for visual specialization in advanced L2 Chinese learners

**DOI:** 10.3389/fnhum.2024.1392788

**Published:** 2024-08-29

**Authors:** Yuxin Hao, Jiawen Guo, Hong Zhu, Bing Bai

**Affiliations:** ^1^Institute of Chinese Language and Culture Education, Huaqiao University, Xiamen, China; ^2^Chinese Language and Culture College, Huaqiao University, Xiamen, China; ^3^Jilin Railway Technology College, Jilin, China; ^4^College of Foreign Languages and Cultures, Xiamen University, Xiamen, China

**Keywords:** Chinese characters, N170, visual specialization, L2Ls, high proficiency

## Abstract

Visual word recognition is crucial for improving reading skills in second language learners (L2Ls). It is unclear whether L2Ls who are native speakers of languages that use alphabetic scripts can recognize Chinese characters at an early stage of visual processing and if their visual specialization can reach a level of word recognition comparable to that of native Chinese speakers. This study aims to uncover the visual specialization mechanism of Chinese L2Ls. A delayed-color matching task was carried out with participants who were Chinese first language speakers (L1Ss) and advanced Chinese L2Ls with Indonesian as their first language. The results of the event-related potentials (ERPs) indicated that L2Ls exhibited significant visual specialization with a predominant distribution of the left-lateralized N170, along with some activation in the right hemisphere. These findings suggest that the early processing stage of Chinese characters by advanced L2Ls is similar to that of adult native speakers, although it is still influenced by their first language and its writing system.

## 1 Introduction

The development of reading skills in second language (L2) learners who are exposed to different writing systems poses a significant challenge due to the need to integrate the cognitive abilities involved in both higher-level processes (such as proficient language use, reading strategies, conceptual understanding, and background knowledge) and lower-level processes like word recognition, which includes phonological and orthographic processing. Numerous studies have demonstrated that L2 reading can be influenced by various factors, including the age of acquisition, task requirements, and level of L2 proficiency (for more information, see the review by Li et al., 2023; Tatsuno and Sakai, 2005). A longstanding area of interest in research on L2 reading is whether the neural mechanism remains consistent between first language speakers (L1Ss) and second language learners (L2Ls), or if it is influenced by the characteristics of different writing systems.

The Chinese writing system,^1^ a logographic system, is significantly different from alphabetic systems such as English and Indonesian, because there is essentially no strict grapheme-to-phoneme mapping in Mandarin (Chen and Shu, 2001; Tan and Perfetti, 1998). One of the most learner-friendly features of the Indonesian writing system is its phonetic consistency. Each letter in the Indonesian alphabet generally corresponds to a specific sound, and the spelling closely matches the pronunciation. This makes reading and writing in Indonesian relatively easier compared to English, where phonetic inconsistencies are more common. In addition, each letter in the word “cat” in English and the word “buku” (meaning “book”) in Indonesian directly corresponds to its phonetic representation. In contrast, the visual form of Chinese characters (such as “猫” for “cat” or “书” for “book”) does not provide direct information about pronunciation. Thus, for L1 speakers of alphabetic languages to read Chinese characters requires high demands on visuospatial processing and relies heavily on memorizing the relationships between visual forms and their pronunciation or meanings (Ge et al., 2006; Huang et al., 2004; Tan et al., 2005).

The distinct properties of writing systems have been widely supported by unique brain activation patterns. For instance, when reading alphabetic words, the left inferior frontal lobe and left temporal-occipitoparietal junction are activated. On the other hand, reading Chinese characters activates the left lateral middle frontal cortex and other regions in the right hemisphere, such as the inferior occipitotemporal cortex (Bolger et al., 2005; Tan et al., 2001, 2005). This dual-sided effect suggests that visual areas in the right hemisphere may help in the spatial processing necessary for the layout of Chinese characters. A recent study by Zhang et al. (2023) found that despite significant differences in orthographic features between Chinese and Mongolian, the event-related potential (ERP) of lexical items in both languages differed substantially from pseudowords in the parietooccipital scalp region. This suggests that a similar neural mechanism is involved in early word processing across different writing systems, consistent with prior research (Chee et al., 2000; Yang et al., 2011).

Word recognition has long been considered a strong indicator of L2 reading development. According to the Convergence hypothesis (Green, 2003), the brain network of L2 learners gradually aligns with that of L1 learners as proficiency in the L2 language increases. Several studies have provided evidence supporting a positive correlation between the level of L2 proficiency and the operation of a shared neural network (Cao et al., 2013; Hernandez et al., 2007; Yang et al., 2011). For instance, high-proficiency L2 learners are more efficient in word recognition compared to low-proficiency L2 learners (Stein et al., 2009) (see review by Cao et al., 2022). While many empirical studies have explored the impact of proficiency on L2 reading (Cao et al., 2013; Chee et al., 2000; Liu et al., 2006; Stein et al., 2009; for a comprehensive review of recent research, see Cao et al., 2022), few studies have delved into the real-time visual specialization of advanced L2 learners of Chinese whose native language is alphabetic.

This study aims to investigate whether advanced L2 learners process different writing systems in overlapping or separate cerebral systems during real-time word recognition. Specifically, the study seeks to determine if advanced L2 learners of Chinese, with Indonesian as their L1 (an alphabetic language), can achieve native-like word recognition. To address this issue, this study uses online measures of brain activation with high temporal resolution to decipher real-time brain responses to stimulus presentation. Previous studies showed that as the brain starts retrieving lexical and semantic information simultaneously (Hauk et al., 2012), the N170 (a negative ERP component with a peak latency of approximately 140–180 ms post-stimulus) becomes indicative of word recognition (Bentin et al., 1999; Maurer et al., 2006; Rossion et al., 2003). Additionally, a plethora of studies have shown that when processing Chinese scripts, the N170 exhibits greater left-lateralization compared to right-lateralization (Yum et al., 2014; Zhao et al., 2012).

The next section begins with an introduction to visual specialization and the main ERP components relevant to word recognition, followed by an overview of experimental studies, especially ERP studies, on the impact of L2 proficiency on Chinese word recognition, concluding with the current ERP study.

## 2 Visual specialization and the main ERPs

The first step in word recognition includes the visual analysis of the printed script, characters, and layout. While processing specific forms, the human brain can differentiate human faces or visual words from a series of visual stimuli. This ability is known as visual specialization and is indicated by the N170 (e.g., Maurer et al., 2005; Rossion et al., 2003; Schendan et al., 1998). Current findings related to the N170 in visual perception mainly fit into two categories.

First, when compared with non-orthographic stimuli, word stimuli trigger enhanced amplitude and lateralized N170 in the left hemisphere (Fu et al., 2012; Kim et al., 2004; Kim and Kim, 2006; Maurer et al., 2006). Rossion et al. (2003) utilized pictures of faces, objects (cars), and English words to investigate the human brain’s ability to categorize objects in the early stages of visual processing, finding that the N170 induced by words significantly differed from that induced by objects, with words eliciting a left-lateralized N170 and objects eliciting a bilateral one. Fu et al. (2012) observed that faces elicited a larger and later N170 response compared to characters, with characters showing a more left-lateralized N170 response. To explore the N170 response to characters across different hemispheres, Zhao et al. (2012) asked participants to determine whether the colors of four types of stimuli (Chinese characters, cartoon faces, line drawings, and stroke combinations) matched those in the previous grid display. Their findings indicated that in the left hemisphere, the N170 response to Chinese characters was significantly stronger than that to other types of stimuli. However, there was no notable difference in response to Chinese characters compared to non-orthographic stimuli, except for line drawings. Conversely, in the right hemisphere, the N170 responses to Chinese characters did not significantly differ from those to non-orthographic stimuli, with the exception of line drawings. These findings suggest that the heightened N170 response to Chinese characters in the left hemisphere could serve as an indicator of proficiency in reading Chinese.

Besides, the amplitude and left-lateralization of the N170 reflect expertise in reading scripts. Alphabetic scripts systematically elicit left-lateralized N170 responses in skilled readers (Maurer et al., 2005), while the N170 has been reported to be left-lateralized in response to logographic stimuli such as Chinese characters (Cao et al., 2011; Lu et al., 2011; Maurer et al., 2008; Yum et al., 2018), and also as showing a bilateral or right-lateralized distribution (Xue et al., 2008; Yum et al., 2011). Wong et al. (2005) conducted a recall judgment task in which both English monolinguals and Chinese-English bilinguals were asked to determine if the presented character matched the previous one or if the underlined character in the current string was a repeat from the previous string. The results showed that Chinese-English bilinguals had enhanced N170 responses for both Roman alphabets and Chinese characters compared to pseudofonts, whereas English monolinguals displayed a greater N170 amplitude for Roman letters compared to Chinese characters and pseudofonts. This mixed lateralization of N170 may be due to varying task demands (Hauk et al., 2012).

To investigate the commonality and particularity of brain activation by different writing systems in bilingual individuals, Kim & Kim (2006) explored differences and similarities among brain activation during visual perceptions of Korean, English, and Chinese words, wherein participants were native Korean speakers who had received more than 6 years of education in both Chinese and English. All Chinese used in their design were one-character Chinese, and the Chinese and Korean words had the same pronunciation as well as the same meaning. The reaction time data showed that the average response time was the shortest for Korean, followed by English, and the longest for Chinese, with significant differences observed only in the comparison between Korean and Chinese. They also found a left-lateralized N170 when Korean and English words were processed, but the left-lateralization was not significant for Chinese characters. In comparison, Yum et al. (2011) found that when processing their native language, both English L1 speakers and Chinese L1 speakers showed a left-lateralized N170 response, while English monolinguals exhibited a right-lateralized N170 when processing Chinese characters.

In addition to the N170 component, the ERP component P100 peaks at approximately 100 ms after the stimulus and is immediately followed by the N170 effect, which helps us determine and extract the time window of early lexical processing. The P100 component indicates cortex activity in response to orthographically atypical objects in the occipitotemporal area. It is considered the onset of the N170 effect (Hauk et al., 2006; Takamiya et al., 2020; Yum et al., 2015). The amplitude of the P100 can be influenced by physical features of visual stimuli (e.g., contrast, luminance, size) and age (Brem et al., 2006), as discussed in the review by Tong et al. (2016). Yum et al. (2018) observed a left-lateralized P100 for native Cantonese spearkers and a right-lateralized P100 for intermediate traditional Chinese learners during the detection of radical position violations (Radicals refer to recurrent subcharacter components), suggesting that the detection of radical position violations may involve global character structure representations in intermediate L2Ls. The variations in the L1 background of the participants in Yum et al. (2018) may have confounded the existing results.

## 3 The high proficiency of L2 and word recognition: ERPs evidence

Perfetti and Liu (2005) and Perfetti (2007) argued that the activation pattern in the brain when learning a new language with a different writing system can be explained by accommodation (where individuals use existing reading network procedures in acquiring a new writing system) and assimilation (where new procedures are used to read in the new writing system by individuals). The differences in design principles between the Chinese writing system and alphabetic scripts in terms of orthography-to-phonology mapping have long fascinated researchers.

To date, the correlation between accommodation/assimilation and L2 proficiency remains mixed in L2 Chinese reading, with some studies favoring accommodation (e.g., Chinese-English, Cao et al., 2011) and others favoring assimilation (e.g., Chinese-English, Deng et al., 2008) (for a review of recent research, see Sun et al., 2015). These results suggest the need for further investigation into whether advanced L2 learners, whose native language is alphabetic, recognize Chinese characters at an early stage of lexical processing and whether they recruit the same brain activation patterns as native Chinese speakers. Meanwhile, it was found that English words, Chinese pinyin (an alphabetic Chinese script), and Chinese characters use the same brain network in both orthographic search and semantic classification tasks (Ding et al., 2003). Accommodation is further supported by the fact that late and early Chinese-English bilinguals use the same network when processing Chinese and English (Chee et al., 2000; Yang et al., 2011), as well as by multilanguage contrast studies (e.g., Spanish, English, Hebrew, and Chinese in Rueckl et al., 2015).

In comparison, assimilation also holds theoretical significance (Leonard et al., 2010). Liu and Perfetti (2003) observed that the peak latencies of the N150 and N250 were earlier for Chinese words than for English words. They also noted that English words (L2) caused reduced activity in the right visual cortex compared to Chinese characters (L1). Cao et al. (2011) found that high proficiency in late English–Chinese bilinguals was associated with increased brain activation in three regions (the right superior parietal lobule, the right lingual gyrus, and the left precentral gyrus) in lexical decision tasks, but this correlation did not appear when writing in pinyin. Therefore, exploration of brain activation patterns during word recognition across different writing systems, as well as the impact of proficiency on the utilization of brain regions in bilingual reading, is necessary.

Based on the studies discussed above, the inconsistency in their results primarily stems from uncontrolled factors that affect the processing of Chinese scripts. Firstly, word recognition efficiency and accuracy can be impacted by previous exposure. Only a few studies (Yum et al., 2011) included participants who were native non-logographic speakers (e.g., native English speakers) with no prior exposure to Chinese, leaving the relationship between N170 lateralization and language proficiency unclear. Secondly, the early stages of visual word recognition can be significantly influenced by the writing system and task demands. In alphabetic scripts, grapheme-phoneme mapping automatically activates and takes precedence over other processing methods like semantic processing.

On the other hand, orthography-to-phonology mapping in Chinese is less transparent, requiring specific attention for other processes to activate (Zhao et al., 2012). Participants engaged in explicit reading and semantic tasks may introduce phonological and semantic activation, potentially affecting early visual processing (Strijkers et al., 2015; Wang and Maurer, 2017). According to Perfetti and Tan (1998), the order of access to lexical information in Chinese character identification starts with the graphic, then proceeds to the phonological, and finally to the semantic. Zhao et al. (2012) argued that the lateralization of N170 may be more influenced by top-down task modulation for Chinese scripts compared to alphabetic scripts. So far, a study using an implicit lexical decision task has not been conducted on the recognition of Chinese characters by L2Ls of Mandarin, but this approach could minimize the influence of phonological and semantic awareness activation.

The current study aimed to investigate the early-stage visual word recognition in Chinese L2 learners whose native languages use alphabetic scripts, through a delayed-color matching task. Two empirical predictions were made below:

**Prediction 1:** high-level L2Ls have the potential to reach proficiency levels equivalent to those of native speakers in the early stages of Chinese character processing. Specifically, we predict that the P100 and N170 responses evoked by Chinese characters will be greater than those evoked by line drawings.

**Prediction 2:** high-level L2Ls may exhibit ERP components that are indistinguishable from those of native adults. Specifically, we anticipate that, for both native speakers and advanced L2Ls, the N170 elicited by Chinese characters in the left hemisphere may be significantly greater than in the right hemisphere, while the N170 component triggered by line drawings may not differ between the hemispheres. Otherwise, the P100 and N170 observed in native speakers may differ from those observed in advanced L2Ls.

## 4 Methods

### 4.1 Participants

We recruited a total of 56 participants, consisting of 27 Chinese native speakers (L1Ss) and 29 advanced-level Chinese learners (L2Ls) with Indonesian as their native language. Due to a limited number of valid trials that met the required criteria, data from two L1Ss and four L2Ls were excluded from the final analysis. Therefore, our final analysis includes data from 25 participants in each group. Participant information for both groups is detailed below:

1. 25 L1Ss, who were native Mandarin speakers (12 males, mean age = 22.5, SD = 2.07, age range = 18 to 24). All L1 participants lived and studied in China with limited exposure to English. They attended fewer than two English classes per week, had no overseas experience, and used English only in formal educational settings.
2. 25 advanced Chinese L2 learners whose first language is Indonesian(12 males; mean age = 23.5; SD = 1.87; ages range = 18 to 26). All L2 participants had studied English and Chinese in Indonesia, starting at the age of 12 or later, making them late learners of both languages and they have taken more Chinese courses than English courses. These participants have lived and studied in Mainland China as international students for a minimum of 4 years. They have attended more than 20 Chinese lessons per week and have successfully passed HSK Level 5.^2^ Chinese was the main language used in their daily lives. They had not taken any English courses while in China and had no experience living in English-speaking countries.

All participants were categorized as right-handed according to the Edinburgh handedness inventory (Oldfield, 1971), and all had normal or corrected vision. None of the participants had a history of reading disability, color blindness, color deficiency, or neurological disease. Informed consent was obtained from all participants before the experiment, and they received payment for their participation. This research was approved by the ethics committee (Ethics Code: M2021014).

### 4.2 Stimuli

We utilized a mixed design incorporating two types of stimuli (Chinese characters and line drawings) and two subject types (L1Ss and L2Ls). The experimental material included 50 non-phonetic compound characters structured from left to right, each consisting of six to nine strokes to reduce the radical position effect (Yum et al., 2018). All characters were presented in the same font to minimize the potential writing effect observed in Yin et al. (2020). These characters were Grade A, the most common Chinese characters in the HSK, which were selected from the *Comprehensive Elementary Chinese* textbook. All stimuli were recognizable, readable, and writable by L2Ls. Additionally, we selected 50 images from Snodgrass and Vanderwart's (1980) line drawings of objects and programmed the experimental materials using E-prime 3.0 software. In total, 50 Chinese characters and 50 line drawings were presented to participants during the study (Table 1).

**Table 1:** Examples of the materials used in the experiment.

Conditions	Examples		
Real characters	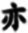	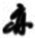	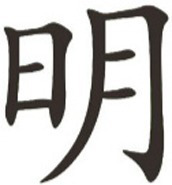
Line drawings	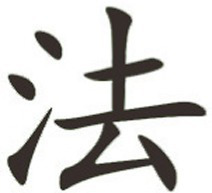	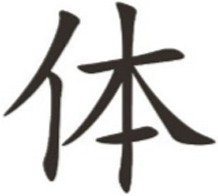	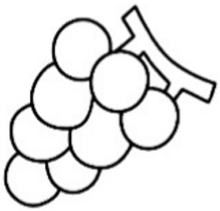

### 4.3 Procedures

**Figure 1:**
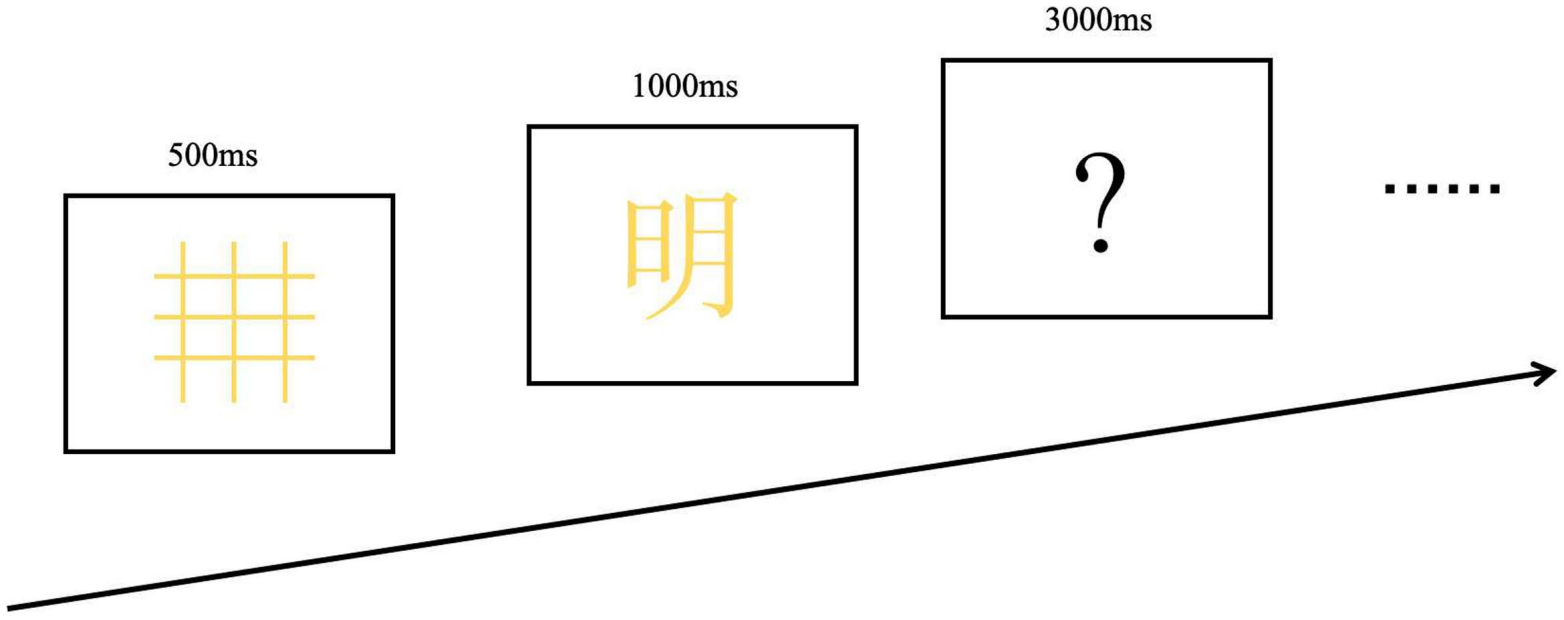
Examples of stimuli and experimental procedures.

The experiment was conducted in a room equipped with sound and electromagnetic shielding. Each participant was seated at a distance of 80 cm from the screen, with a vertical visual angle of 4 degrees. The experimental procedure consisted of the following steps: Initially, a colored grid (red, green, or yellow) appeared in the center of the screen for 500 ms, followed by the presentation of the target stimulus (Chinese characters or line drawings) in one of the three colors for 1,000 ms. Subsequently, a question mark was displayed for 3,000 ms, prompting participants to determine whether the color of the grid matched that of the target stimulus. Participants were instructed to press the “D” key if there was a match and the “K” key if there was no match, with their left and right hands balanced throughout the experiment. After 20 practice trials, all participants completed two blocks of 50 trials each (100 trials in total). Two types of stimuli (Chinese characters and line drawings) were presented in random order with equal probability. Before starting the formal experiment, participants had already become familiar with the experimental procedure through practice sessions (Figure 1).

### 4.4 Statistical analysis of behavioral data

In order to analyze behavioral data, we performed a repeated measures analysis of variance (ANOVA) with two groups (L1Ss vs. L2Ls) and two stimulus types (Chinese characters vs. line drawings) for both accuracy and reaction times. Group was considered the between-subject factor, and stimulus type was considered the within-subject factor. To account for violations of sphericity, the Greenhouse–Geisser correction was applied, and the Bonferroni method was used to adjust for multiple comparisons. A significance level of *α* = 0.05 was set to determine statistical significance.

### 4.5 EEG recordings and preprocessing

Electroencephalography (EEG) signals were continuously recorded during the experiment using 64 Ag/AgCl electrodes. Sixty-two of these electrodes were embedded in an elastic cap (Quick-Cap, Neuromedical Supplies, Sterling, TX), while the remaining two electrodes were placed on the right and left mastoids. All channels were referenced to a channel positioned between the Fz and FCZ and were later referenced offline to the average of the two mastoids. Additionally, a ground electrode was positioned on the forehead anterior to the Fz electrode.

Vertical and horizontal electrooculograms (EOGs) were bipolarly recorded from electrodes located above and below the right eye and on the outer canthi of each eye, respectively. The data were sampled at 1000 Hz, with the interelectrode impedance was kept below 5 kΩ. The data were processed using Curry 8.0 software. EEG activity was filtered offline utilizing a bandpass zero-phase shift filter with a high cutoff of 30 Hz and a low cutoff of 0.05 Hz at 12 dB/oct.

Before analysis, any large artifacts (exceeding ±70 μV) were removed. Participants with data containing over 20 artifacts in any condition were excluded, resulting in 93.60% artifact-free trials for the L1Ss and 91.3% artifact-free trials for the L2Ls. The event-related potential (ERP) was computed for each participant and each electrode within an epoch ranging from −100 ms to 400 ms after the onset of the critical stimulus.

### 4.6 Event-related potential analysis of EEG data

Baseline corrections were applied to the interval of −100 to 0 ms relative to the stimulus onset. Based on visual inspection of the ERP waveforms in the current study and previous studies (Yum and Law, 2021; Zhao et al., 2012), we analyzed the P100 and N170 components of the electrodes in the occipitotemporal region in the time windows of 80–140 ms and 140–200 ms, respectively, with the mean values of P7, PO7, and O1 taken on the left and P8, PO8, and O2 taken on the right.

For each component, we conducted a repeated measures ANOVA with a two-group (L1Ss vs. L2Ls) by two stimulus types (Chinese characters vs. line drawings) by two hemispheres (left vs. right) design on the mean amplitudes of ERP. The group was included as the between-subject factor and stimulus type as the within-subject factor. The Greenhouse–Geisser correction was used to compensate for violations of sphericity, and the Bonferroni method was employed to correct for multiple comparisons. Post-hoc comparisons were conducted to further explore significant main effects and interactions.

Additionally, considering the differences in behavioral accuracy between the two types of stimuli (see section 5.1 Behavioral results), we performed a linear regression analysis to assess the potential impact of these differences on the mean amplitudes of ERP. Behavioral accuracy was included as the independent variable, and the mean amplitudes of ERP were included as the dependent variable. The model fitting was done using the lm package in R software (version 4.1.1).

## 5 Results

### 5.1 Behavioral results

The L1s’ behavioral accuracies for Chinese characters and line drawings were 93.93% (±1.97) and 98.12% (±1.67), respectively. In contrast, the L2Ls’ accuracies were 87.67% (±9.85) for Chinese characters and 90.43% (±10.52) for line drawings. The behavioral reaction times for Chinese characters and line drawings were 327.35 (±110.26) and 328.14 (±112.21), respectively, for the L1s, while for the L2Ls they were 351.88 (±138.30) and 360.11 (±144.97). These results suggest that participants maintained focus throughout the experiment and performed well on the tasks.

A repeated measures ANOVA was conducted on the participants’ accuracy. The main effect of stimulus type (Chinese characters and line drawings) was significant [*F* (1,48) = 66.377, *p* < 0.001, η^2^_p_ = 0.580]; however, the main effect of group was not significant [*F* (1,48) = 2.856, *p* = 0.098], and the interaction between stimulus type and the group was also not significant [*F* (1,48) = 2.194, *p* = 0.145].

Additionally, a repeated measures ANOVA was performed on the participants’ reaction time. The main effect of stimulus type (Chinese characters and line drawings) was not significant [*F* (1,48) = 2.113, *p* = 0.153], the main effect of group was not significant [*F* (1,48) = 1.121, *p* = 0.295], and the interaction between stimulus type and group was also not significant [*F* (1,48) = 0.917, *p* = 0.343].

### 5.2 ERP results

**Figure 2:**
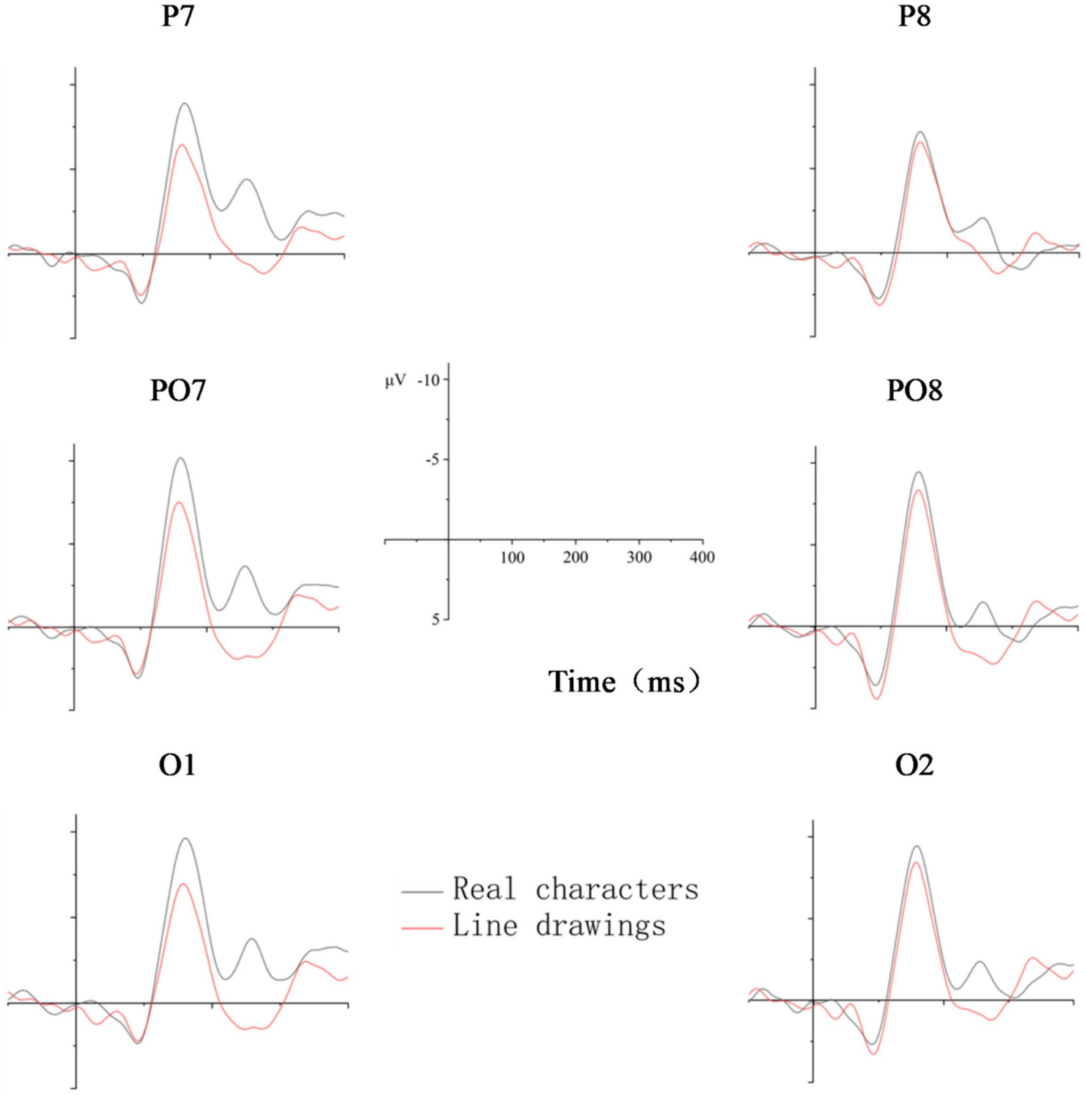
Averaged N170 waveforms for Chinese characters and line drawings among the L1Ss. The X-axis represents the duration, and each hash mark represents 50 ms. The Y-axis represents the voltage, which ranged from −10 to +5 μV. Negativity is plotted upward.

**Figure 3:**
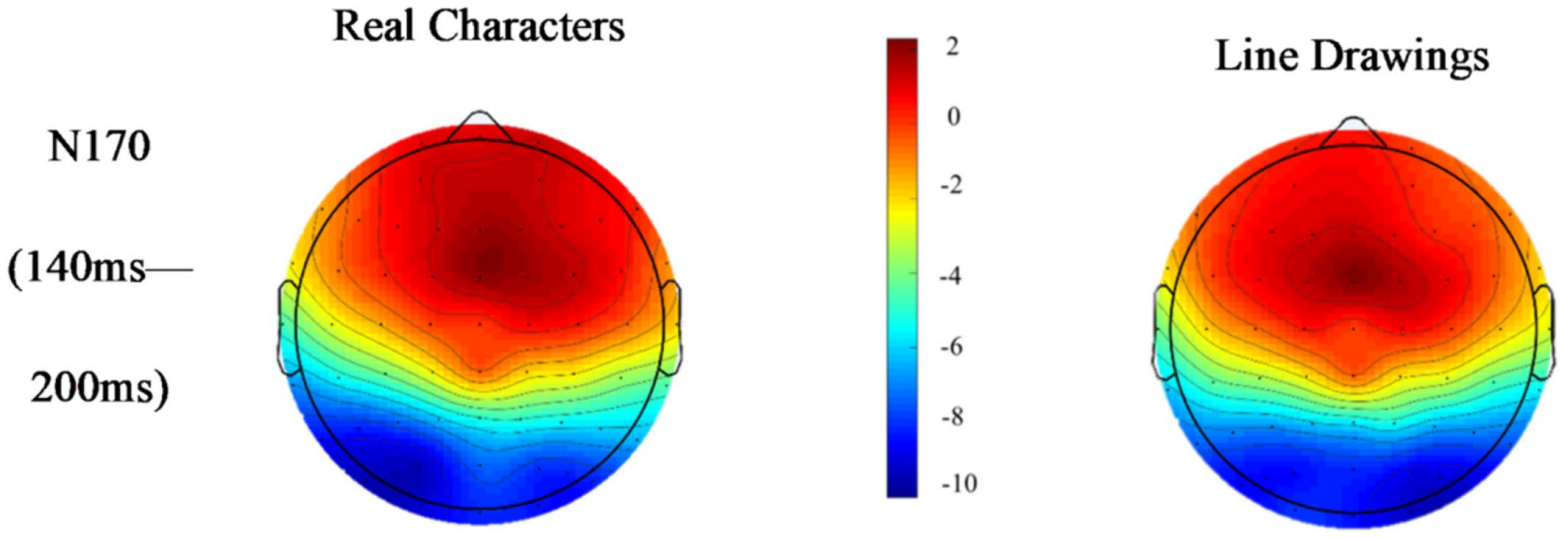
Topographic maps of responses to Chinese characters and line drawings among the L1Ss.

**Figure 4:**
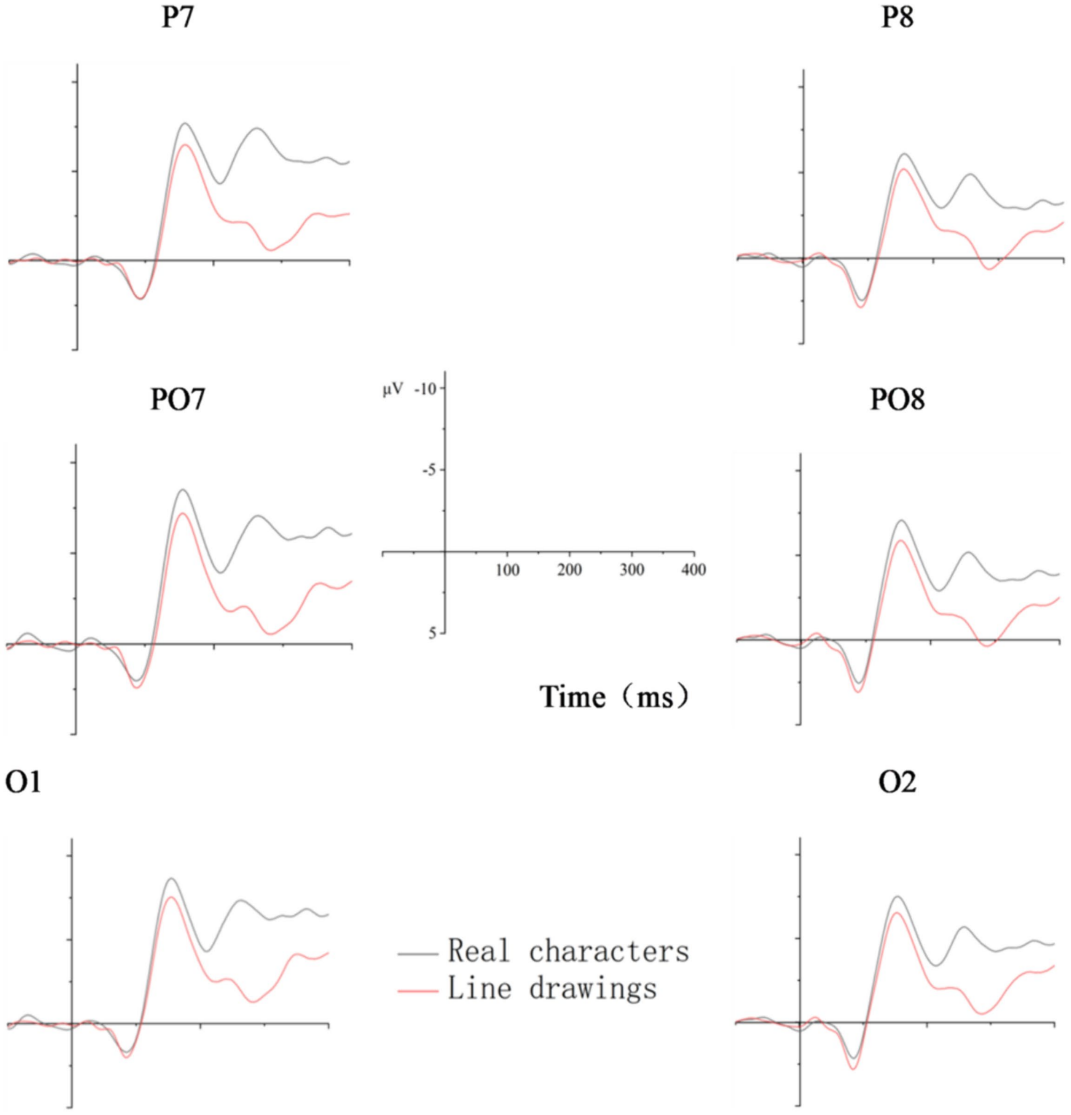
Averaged N170 waveforms for Chinese characters and line drawings among the L2Ls. The X-axis represents the duration, and each hash mark represents 50 ms. The Y-axis represents the voltage, which ranged from −10 to +5 μV. Negativity is plotted upward.

**Figure 5:**
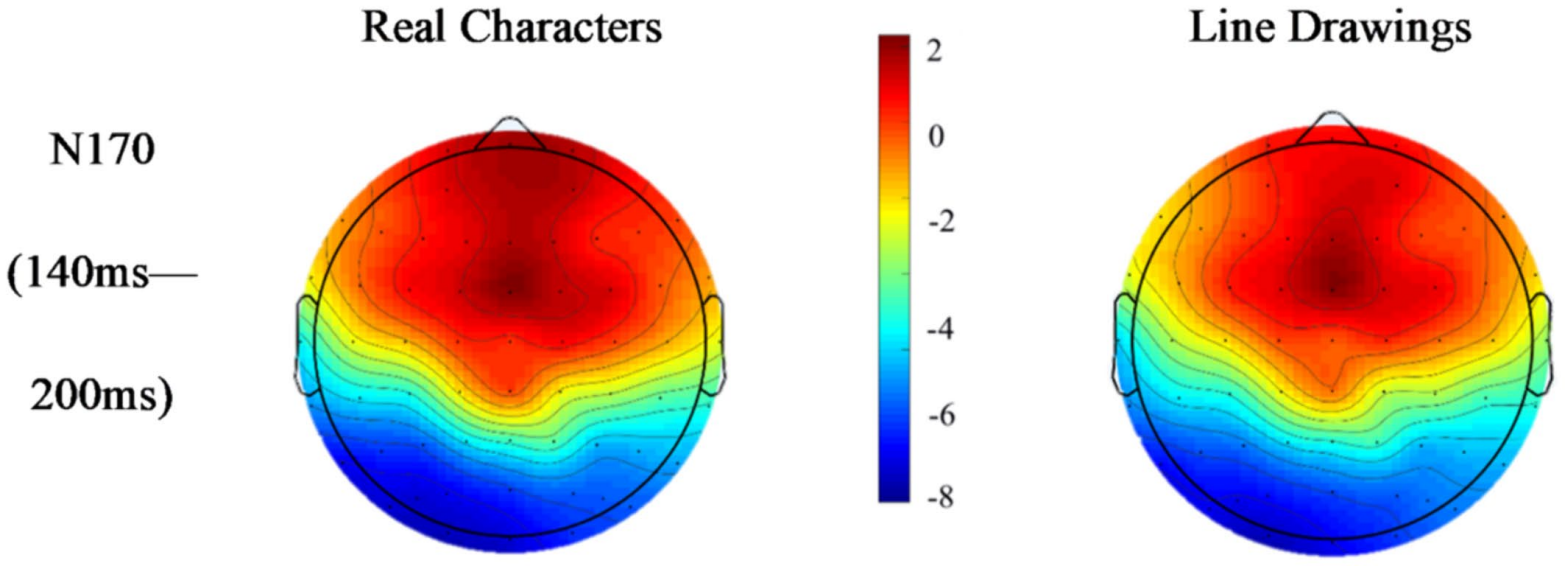
Topographic maps of responses to the Chinese characters and line drawings among the L2Ls.

Figures 2–5 below illustrate that there was nearly complete overlap between Chinese characters and line drawings in the 80–140 ms time window for both L1Ss and L2Ls. Moreover, both groups elicited a noticeable N170 response in the 140–200 ms time frame, with a more pronounced effect observed in the left hemisphere. The *p* values for the regions of interest can be found in Table 2.

**Table 2:** The *p* values of the ROIs.

ERP component	Effects	*p*-value
P100	Stimulus type	0.115
	Hemisphere	0.677
	Group	0.374
	Stimulus type * Hemisphere	0.372
	Stimulus type * Group	0.425
	Group * Hemisphere	0.804
	Stimulus type * Hemisphere * Group	0.503
N170	Stimulus type	<0.001***
	Hemisphere	0.041*
	Group	0.790
	Stimulus type * Hemisphere	<0.001***
	Stimulus type * Group	0.540
	Group * Hemisphere	0.289
	Stimulus type * Hemisphere * Group	0.003*

*p* < 0.10; *p < 0.05; **p < 0.001; ***p < 0.0001.

In the 80–140 ms time window, the main effect of stimulus type (Chinese characters and line drawings) was not significant [*F* (1,48) = 2.57, *p* = 0.115]. The main effect of hemisphere [*F* (1,48) = 0.18, *p* = 0.677], the main effect of group (L1Ss and L2Ls) [*F* (1,48) = 0.81, *p* = 0.374], and all interaction effects were also not significant.

In the 140–200 ms time window, the main effect of stimulus type (Chinese characters and line drawings) was significant [*F* (1, 48) = 18.89, *p* 0.001, η^2^_p_ = 0.28]. The main effect of the hemisphere was also significant [*F* (1, 48) = 4.42, *p* = 0.041, η^2^_p_ = 0.08], but the main effect of the group was not significant [*F* (1, 48) = 0.07, *p* = 0.790]. The interaction between group and stimulus type was not significant [*F* (1, 48) = 0.38, *p* = 0.54], nor was the interaction between group and hemisphere [*F* (1, 48) = 1.15, *p* = 0.289]. The interaction effect between stimulus type and hemisphere was significant [*F* (1, 48) =15.24, *p* < 0.001, η^2^_p_ = 0.24]. More importantly, the group X stimulus type X hemisphere interaction was significant [*F* (1, 48) = 9.86, *p* = 0.003, η^2^_p_ = 0.17].

As for prediction 1, we compared the average wave amplitudes produced by the same type of stimuli (Chinese characters and line drawings) in the left and right hemispheres for L1Ss and L2Ls, respectively. Tests of the effects of stimulus type within each group showed that for L1Ss, the mean amplitude of the ERP in response to Chinese characters in the left hemisphere (−7.462 μV) was more negative than in the right hemisphere (−6.363 μV) [*F* (1,48) = 4.19, *p* = 0.046, η^2^_p_ = 0.081], while the difference between the left and right hemispheres for line drawings was not significant [*F* (1,48) = 0.04, *p* = 0.532]. On the other hand, for L2Ls, the difference in mean wave amplitude induced by Chinese characters in the left (−6.895 μV) and right hemispheres (−5.649 μV) was significant [*F* (1,48) = 5.38, *p* = 0.025, η^2^_p_ = 0.101], but there was no significant difference in line drawings between the left and right hemispheres [*F* (1,48) = 4.03, *p* = 0.061].

As for prediction 2, we compared the mean wave amplitudes evoked by Chinese characters and line drawings in the same hemisphere (left and right hemispheres, respectively) for L1Ss and L2Ls. The tests of the effects of hemisphere within each stimulus type level revealed that for L1Ss, in the left hemisphere the difference between Chinese characters (−7.46 μV) and line drawings (−5.209) was significant [*F* (1,48) = 25.12, *p* < 0.001, η^2^_p_ = 0.344], but there was no significant difference between the two stimuli in the right hemisphere [*F* (1,48) = 2.99, *p* = 0.090]. For L2Ls, in the left hemisphere, the difference between Chinese characters and line drawings was significant [*F* (1,48) = 7.49, *p* = 0.009, η^2^_p_ = 0.135], with the amplitude of Chinese characters (−6.89 μV) significantly greater than that of line drawings (−5.664 μV); in the right hemisphere, the amplitude of Chinese characters (−5.649 μV) was significantly more negative than that of line drawings (−4.575 μV) [*F* (1,48) = 5.21, *p* = 0.027, η2p = 0.098].

In summary, the ERP results demonstrated that for both L1Ss and L2Ls, the P100 components elicited by Chinese characters and line drawings did not show significant differences. The N170 component evoked by Chinese characters was significantly larger than that elicited by line drawings in the left hemisphere for both groups. Additionally, the N170 amplitude evoked by Chinese characters was significantly more negative than that evoked by line drawings in the right hemisphere for L2Ls, whereas no such difference was observed for L1Ss.

### 5.3 Regression analysis

Linear regression analysis showed that the relationship between behavioral accuracies and ERP was not significant [*β* = 0.029, SE = 0.080, *t* = 0.357, *p* = 0.722; 95% CI = (−0.130, 0.187)]. The model’s *R*^2^ value was 0.001, indicating that behavioral accuracy could only explain 0.13% of the variation in ERP. The results of the F-test (*F* = 0.127, df = 1, 98, *p* = 0.722) further indicated that the model did not have a significantly improved ability to explain ERP variation compared to a model that only included the intercept.

## 6 Discussion

The current study aimed to investigate the visual specialization of advanced Chinese L2Ls at an early stage by using a delayed color-matching task. The results of the ERP analysis suggest that second language learners can reach a level of visual specialization comparable to that of native speakers. Specifically, second language learners are able to perform rapid and automatic visual specialization of Chinese characters, even with the unique addition of brain activation patterns.

This study provides support for the potential of advanced second language learners (L2Ls) to exhibit native-like visual specialization of Chinese characters, as demonstrated by the brain topology seen in the left-lateralized N170 response to Chinese characters. The left-lateralization of N170 is a significant indicator of visual specialization for words, distinct from the N170 effects seen for faces and objects, which often exhibit bilateral or right-lateralized patterns (Bentin et al., 1999; Maurer et al., 2005; Rossion et al., 2003). Essentially, the left-lateralized N170 represents a specific neural response to words, demonstrating the brain’s unique sensitivity to linguistic stimuli. Just as Yum et al. (2014) found that bilingual learners with high efficiency in vocabulary tasks showed a significant increase in left-lateralized N170, while bilingual learners with low efficiency in vocabulary tasks exhibited a distinct right-lateralized increase in N170 amplitude, our study also reveals that advanced L2Ls who frequently read Chinese characters in their daily lives exhibit a consistently left-lateralized N170 response to Chinese characters, similar to native Chinese speakers. Maurer et al. (2007) proposed that the repeated practice of grapheme-phoneme decoding in visual word recognition during the process of learning to read is responsible for the left-lateralized N170 associated with visual specialization. Therefore, our results suggest that advanced L2Ls are capable of developing grapheme-phoneme mapping in response to characters.

One possible explanation could be the familiarity with the stimuli used, which is a result of significant reading experience, as indicated in previous studies (Cao et al., 2011; Maurer et al., 2008; Tong et al., 2016; Yum and Law, 2021). For instance, Xue et al. (2019) conducted a study comparing the neural mechanisms of native Chinese reading when individuals read Chinese characters and cursive Chinese characters. The results of the ERP showed that only printed characters elicited the left-lateralized N170, while cursive characters evoked a bilateral N170 response. This difference may be attributed to the need for greater familiarity with cursive characters in terms of visual perception. A similar familiarity effect was also noted in L2 reading (Maurer et al., 2008), where Japanese L1 speakers exhibited a left-lateralized N170 when processing Japanese hiragana and katakana,^3^ while English L1 speakers showed a bilateral N170 effect. Therefore, visual familiarity resulting from reading experience and proficiency could also play a role in the left-lateralization of the N170.

Although our findings demonstrate that advanced L2 learners can achieve a level of visual specialization similar to that of L1 speakers, it is crucial to note that their brain topography was not completely identical to that of L1 speakers. In contrast to previous studies (Cao et al., 2011; Maurer et al., 2008; Tong et al., 2016; Yum and Law, 2021), we found that among the L2 learners, the N170 response elicited by Chinese characters in the right hemisphere showed a significant difference compared to that elicited by line drawings, whereas this significant difference was not observed in the L1 speakers. Previous research has shown right-lateralized or bilateral visual expertise effects for both novel objects and faces (Bentin et al., 1999; Rossion et al., 2003; Cao et al., 2011; Maurer et al., 2010). However, we argue that, due to the use of an implicit color-matching task aimed at minimizing top-down modulation in the current study, the L2 learners recognized the Chinese characters through a pre-existing expertise network (McCandliss et al., 2003; Nelson et al., 2009). These findings suggests that the underlying neural mechanism for reading in L2 is still influenced by their first language and its writing system.

The P100 component reflects the early and rapid processing of both simple and complex stimuli, laying the groundwork for visual specialization (Maurer et al., 2005, 2006; Zhao et al., 2014). This component is influenced by top-down modulation, with variations in latency and amplitude depending on the research paradigm and task requirements (Segalowitz and Zheng, 2009; Taylor, 2002). The absence of top-down modulation may account for the results obtained in our current study. Unlike previous studies (Segalowitz and Zheng, 2009; Yum et al., 2018), we conducted a delayed color-matching task and found no significant differences in P100 between Chinese characters and line drawings. This difference may be due to variations in research paradigms, as the delayed color-matching task reduces the impact of top-down processes on a variety of stimuli and diminishes the potential for phonological and semantic activation (Zhao et al., 2012).

Interestingly, after a stimulus presentation of 200 ms, the N250 evoked by Chinese characters was greater than that evoked by line drawings for both the L1s and the L2s. The N250 reflects the mapping of prelexical formal representations to orthographic representations (Holcomb and Grainger, 2006). These findings inspire us to focus on the orthographic processing of Chinese learners in our future research.

## 7 Conclusion and limitation

This study discovered that advanced Chinese L2 learners with native backgrounds in an alphabetic script exhibit significant visual specialization and a predominant distribution of left-lateralization, with some activation in the right hemisphere. The results suggested that the spatiotemporal cortical activation of advanced L2 learners is similar to that of Chinese native speakers, although the influence of their pre-existing expertise network (first language and its writing system) persists.

However, this research can be further explored. Firstly, this study focused solely on the real-time word recognition process of advanced Chinese L2 learners without comparing the mechanisms involved among learners at different proficiency levels. Secondly, this study only included native speakers of alphabetic languages, but did not compare the neural mechanisms underlying their reading of Chinese characters with those of L2 learners whose first languages have varied logographic systems. Thirdly, while the experiment controlled for readability and familiarity of the linguistic stimuli, it did not investigate the potential impact of unfamiliar characters on the early stage of lexical decisions in advanced L2 learners.

## Data Availability

The raw data supporting the conclusions of this article will be made available by the authors, without undue reservation.

## Footnotes

^1^In the modern Chinese writing system, the writing form includes regular characters and cursive characters. Regular characters (e.g., ) are the most used and widely recognized form of written Chinese, while cursive characters (e.g., ) are less commonly used, only employed in limited situations (e.g., billboards and trademarks), but still recognizable and understandable for skilled Chinese readers. In this study, the writing system of Chinese exclusively refers to the simplified writing system of Mandarin.

^2^The Chinese Proficiency Test (HSK) is a standardized international Chinese proficiency test for non-Chinese native speakers (including foreigners, overseas Chinese, Chinese Americans and Chinese ethnic minorities). Level 5 corresponds to Level 5 of The Chinese Language Proficiency Scales for Speakers of Other Languages and Level C1 of the Common European Framework of Reference for Languages (CEF). Candidates who pass HSK (Level 5) can read Chinese newspapers and magazines, enjoy Chinese movies and TV programs, and give complete speeches in Chinese.

^3^Japanese Hiragana and Katakana are two components of the three writing systems used in Japanese, collectively known as Kana. Hiragana is a phonetic alphabet used primarily for native Japanese words and grammatical elements. Katakana, on the other hand, is mainly employed for foreign loanwords (non-native Japanese terms), foreign names and places, onomatopoeia, as well as technical and scientific terminology. In addition, the Japanese writing system also includes Kanji, which are characters borrowed from Chinese.
